# Supplementary material for: Stage-adaptive integration of polydopamine promotes human pluripotent stem cell-derived alveolar organoids differentiation and maturation
Source: Mater Today Bio. 2026 Jun 18;39:103362. doi: 10.1016/j.mtbio.2026.103362 (PMC13315959; doi:10.1016/j.mtbio.2026.103362)
Supplement: Multimedia component 1 [file mmc1.docx]

**Supplementary information**

Stage-adaptive integration of polydopamine promotes hPSC-derived alveolar organoids differentiation and maturation

*Ruihao Lan^#^, Yu Chen, Zhiying Liao, Hengrui Zhang, Caidie Zhong, Jiaxiang Yin, Chang Du, Tao Xu, Hao Meng^#*^ and Huisheng Liu^*^*

This file includes:

Supplementary Figures 1 to 8

Supplementary Tables 1 to 4


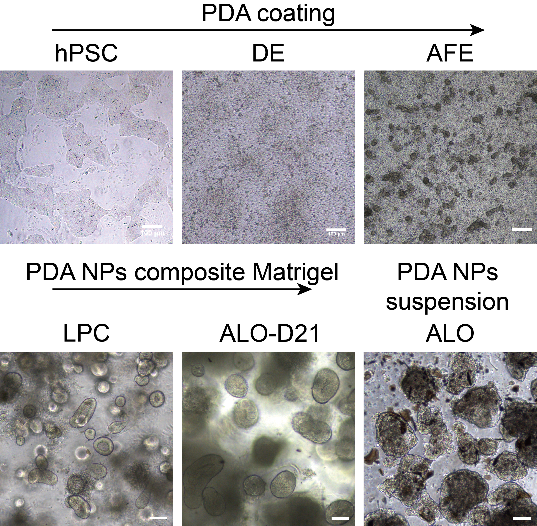


Fig.S1 Representative bright-field images of hPSC derived ALO differentiation. There are three culture formats during hPSC-ALO differentiation, including 2D monolayer culture (with PDA coating), Matrigel 3D culture (with PDA NPs mixed in Matrigel), suspended culture (with PDA NPs suspension in medium), scale bar: 100 µm.


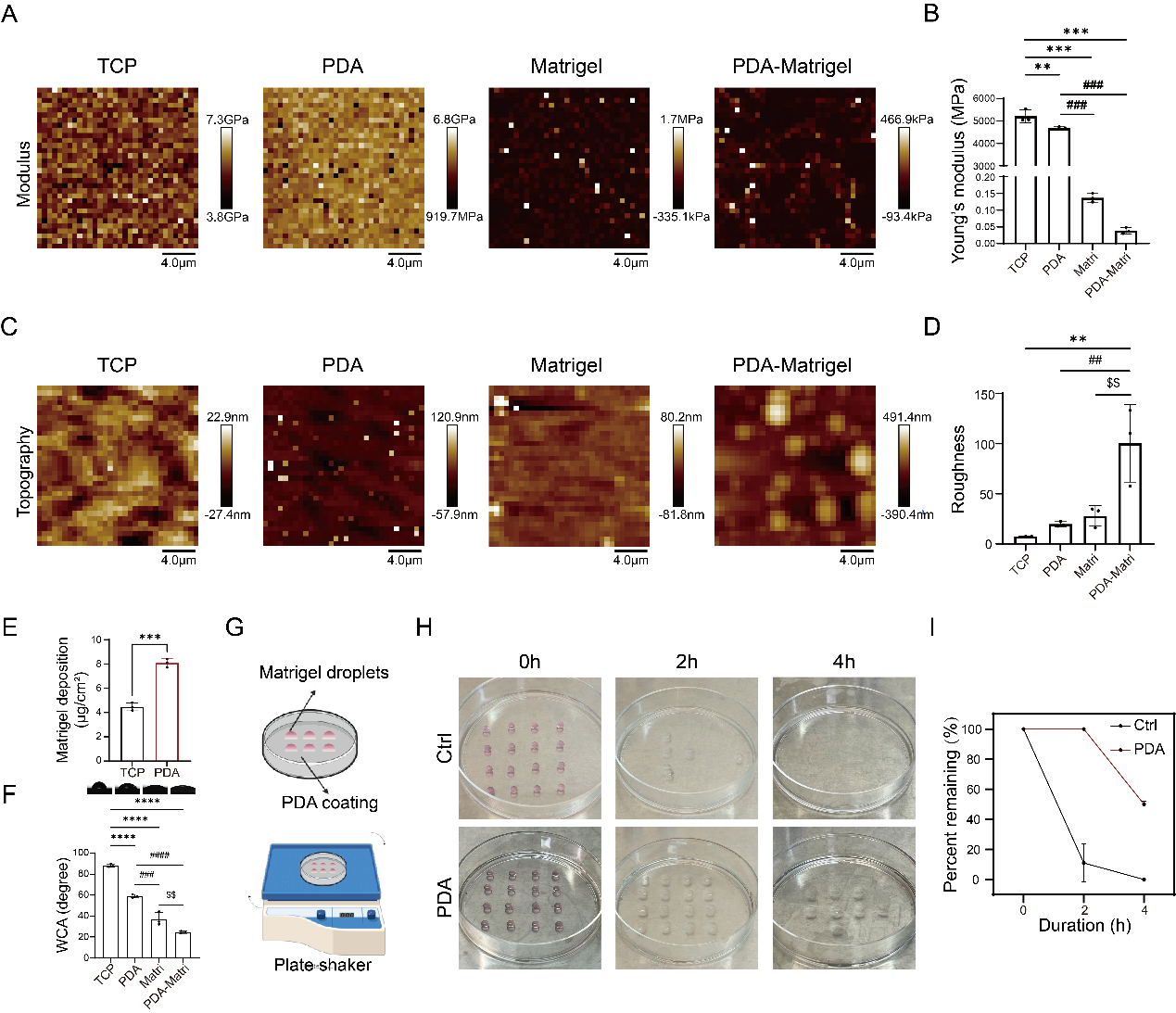


Fig.S2 Physiochemical characterization of PDA coating. A) Representative AFM maps displaying the spatial distribution of the Young’s modulus on tissue culture plate (TCP), PDA-coated TCP (PDA), Matrigel-coated TCP (Matrigel), and PDA-Matrigel-coated TCP (PDA-Matrigel), scale bar: 4 µm; B) Quantification of AFM-derived Young’s modulus for the indicated substrates; C) Representative AFM topography (height) of TCP, PDA, Matrigel and PDA-Matrigel, scale bar: 4 µm; D) Quantification of surface roughness derived from AFM topography; E) Comparison of protein deposition between TCP and PDA; F) Water contact angle (WCA) measurements evaluating surface wettability of TCP, PDA, Matrigel and PDA-Matrigel; G) Schematic illustration of the Matrigel droplet adhesion assay: an array of Matrigel droplets was deposited onto Ctrl or PDA-coated plates and subjected to orbital shaking; H) Representative images of Matrigel droplets retention on Ctrl and PDA substrates after agitation for 0, 2, and 4 h; I) Quantification of the percentage of Matrigel droplets remaining over time during agitation. Statistical significance is indicated as follow: ** and $$ *P* < 0.01, *** and ### *P* < 0.001; * indicates comparisons with TCP; # indicates comparisons with PDA; $ indicates comparison with Matri.


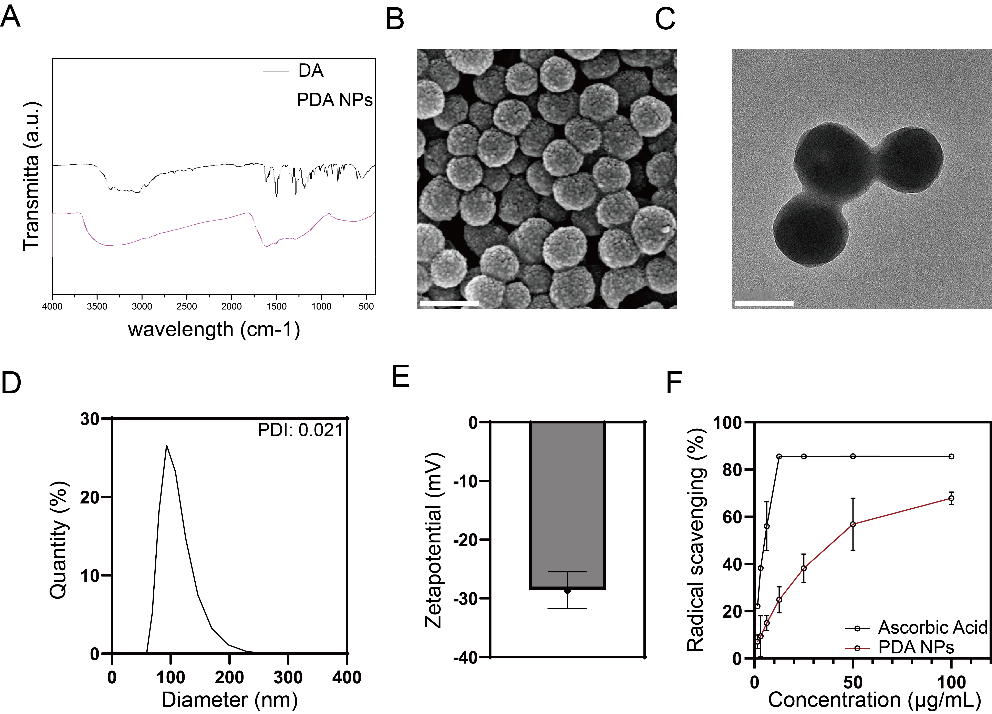


Fig.S3 PDA nanoparticles characteristics. A) Fourier-transform infrared (FTIR) spectra of dopamine and PDA NPs; B) Representative SEM image of PDA NPs, scale bar: 200 nm; C) Representative TEM image of PDA NPs, scale bar: 100 nm; D) DLS analysis of particle size distribution of PDA NPs (PDI: 0.021); E) Zeta potential of PDA NPs measured in aqueous suspension; F) DPPH radical scavenging activity of ascorbic acid and PDA NPs at different concentrations.


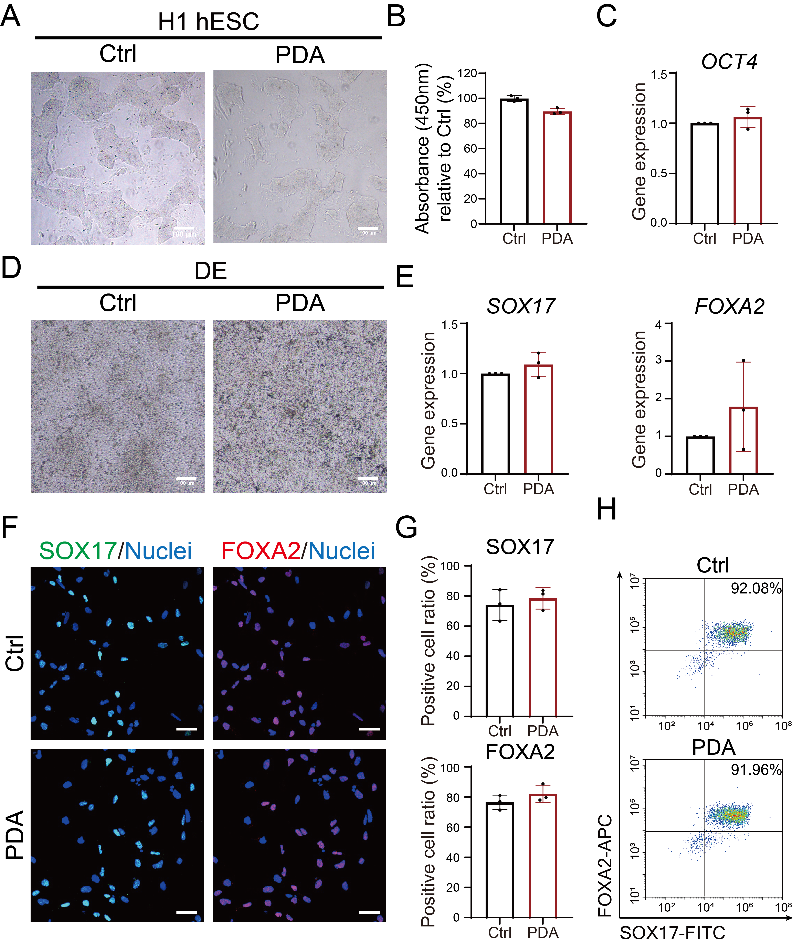


Fig.S4 PDA coating maintains H1 hESC proliferation and DE differentiation. A) Representative bright-field images of H1 hESC cultured on Ctrl and PDA-coated substrates, scale bar: 100 µm; B) CCK-8 assay of H1 hESC viability on Ctrl versus PDA-coated substrates; C) qPCR analysis of the *OCT4* expression of hPSC cultured on Ctrl and PDA-coated substrates; D) Representative bright-field images of DE cells differentiated on Ctrl or PDA-coated substrates, scale bar: 100 µm; E) qPCR analysis of DE marker genes *SOX17* and *FOXA2* expression in DE cells generated on Ctrl and PDA-coated substrates; F) Immunofluorescence imaging of SOX17 (green) and FOXA2 (red) , (nuclei: blue; scale bar: 50 µm); G) Quantification of SOX17 positive and FOXA2 positive cell percentages from (F); H) Representative flow cytometry profiles of SOX17 and FOXA2 positive cells in DE.


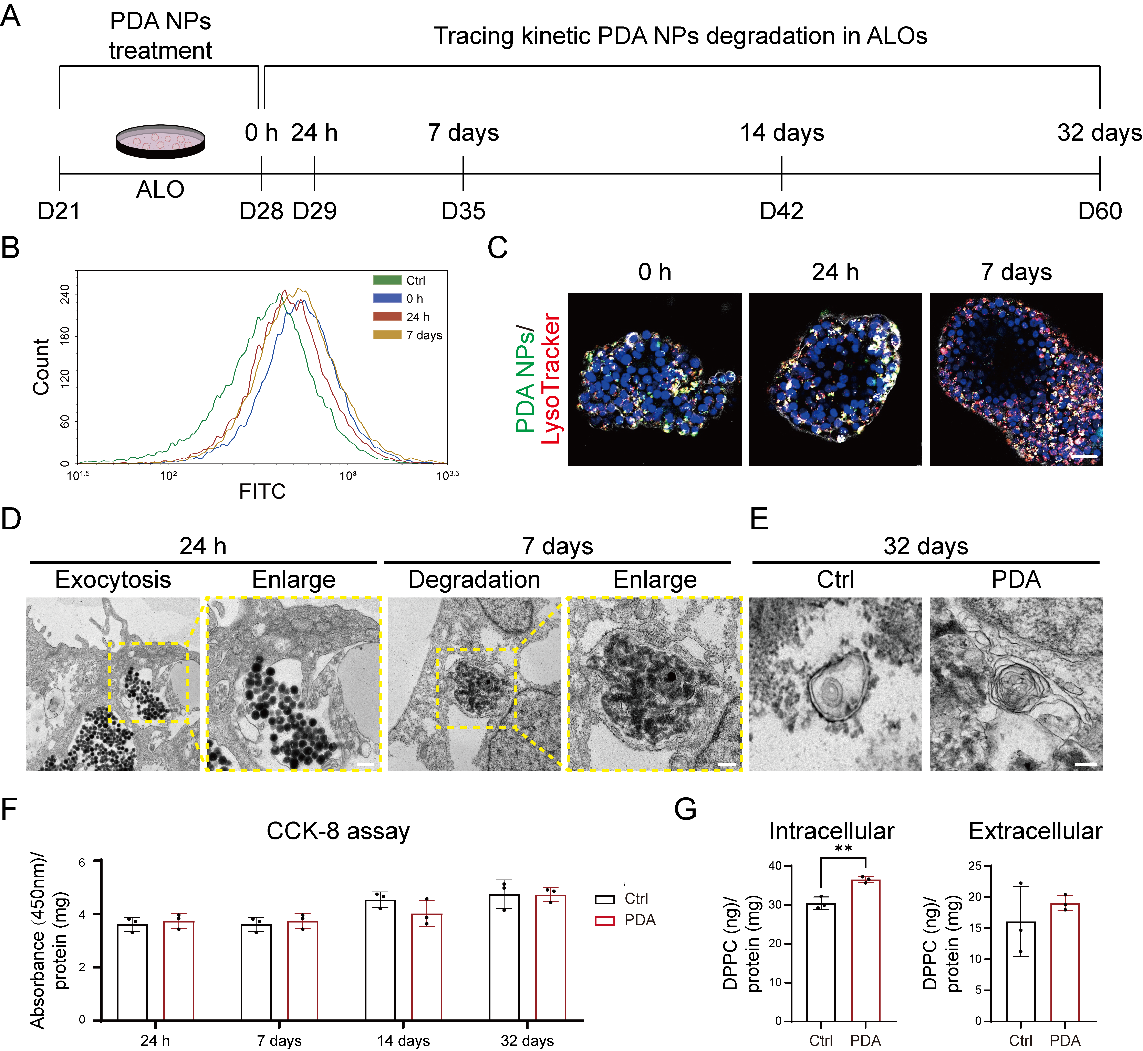


Fig.S5 Evaluation of the degradation and biosafety of PDA NPs. A) Schematic timeline of PDA NP degradation tracing in ALOs; B) Flow cytometry analysis of FITC-labeled PDA NPs in ALOs at 0 h, 24 h, and 7 days, control group (without PDA treatment at 0 h) used as the negative control; C) IF co-staining of FITC-labeled PDA NPs and LysoTracker in ALOs at 0 h, 24 h, and 7 days, scale bar: 50 µm; D) TEM images of ALOs showing membrane-proximal PDA NPs exocytosis at 24 h and PDA debris within lamellar body at 7 days, scale bar: 200 nm; E) TEM images of ALOs after extended culture for 32 days showing lamellar body structures without obvious accumulation of PDA NPs or ultrastructural abnormalities, scale bar: 200 nm; F) CCK-8 assay evaluating the long-term viability of control and PDA-treated ALOs after 24 h (D29), 7 days (D35), 14 days (D42), and 32 days (D60); G) Quantification of intracellular and extracellular DPPC of ALOs. Statistical significance is indicated as follows: ***P* < 0.01.


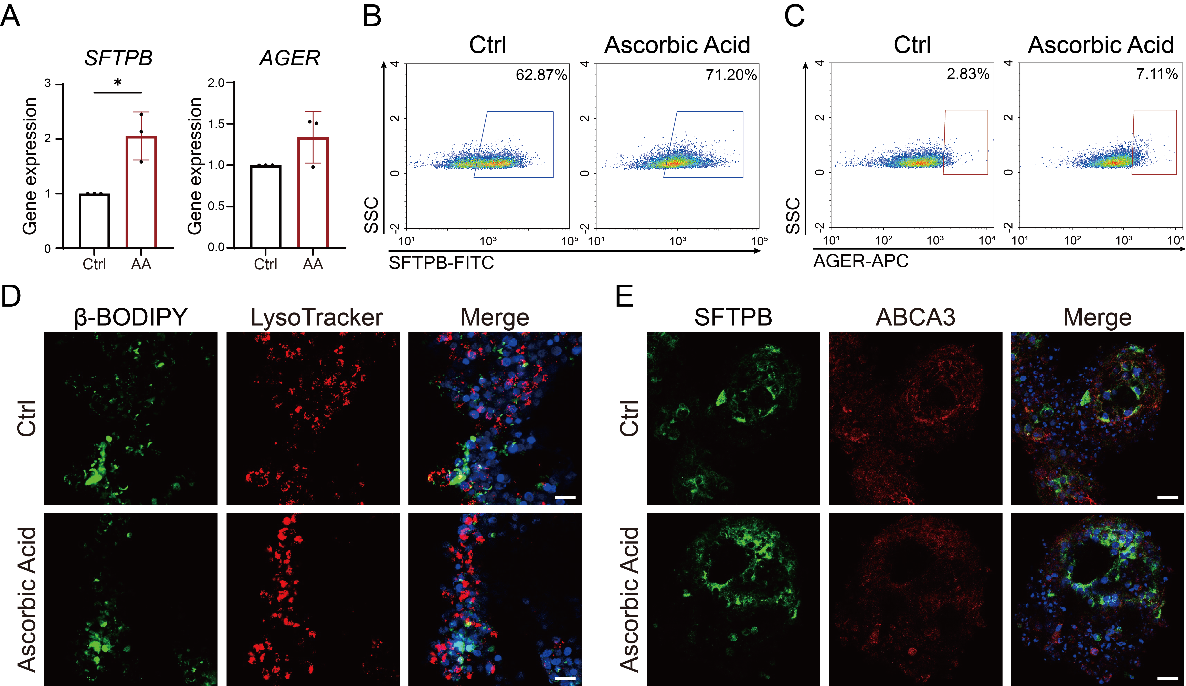


Fig.S6 Ascorbic acid (AA) treatment promotes SFTPB^+^AT2 lineage differentiation but does not fully enhance LB maturation. A) qPCR analysis of *SFTPB* and *AGER* expression in control and AA treated ALOs; B, C) Representative flow cytometry profiles showing the proportions of SFTPB^+^ AT2 cells and AGER^+^ AT1 cells in control and ascorbic acid treated ALOs; D) IF staining of β-BODIPY and LysoTracker in control and ascorbic acid treated ALOs, scale bar: 50 µm; E) IF staining of SFTPB and ABCA3 in control and ascorbic acid treated ALOs, scale bar: 50 µm. Statistical significance is indicated as follows: **P* < 0.05.


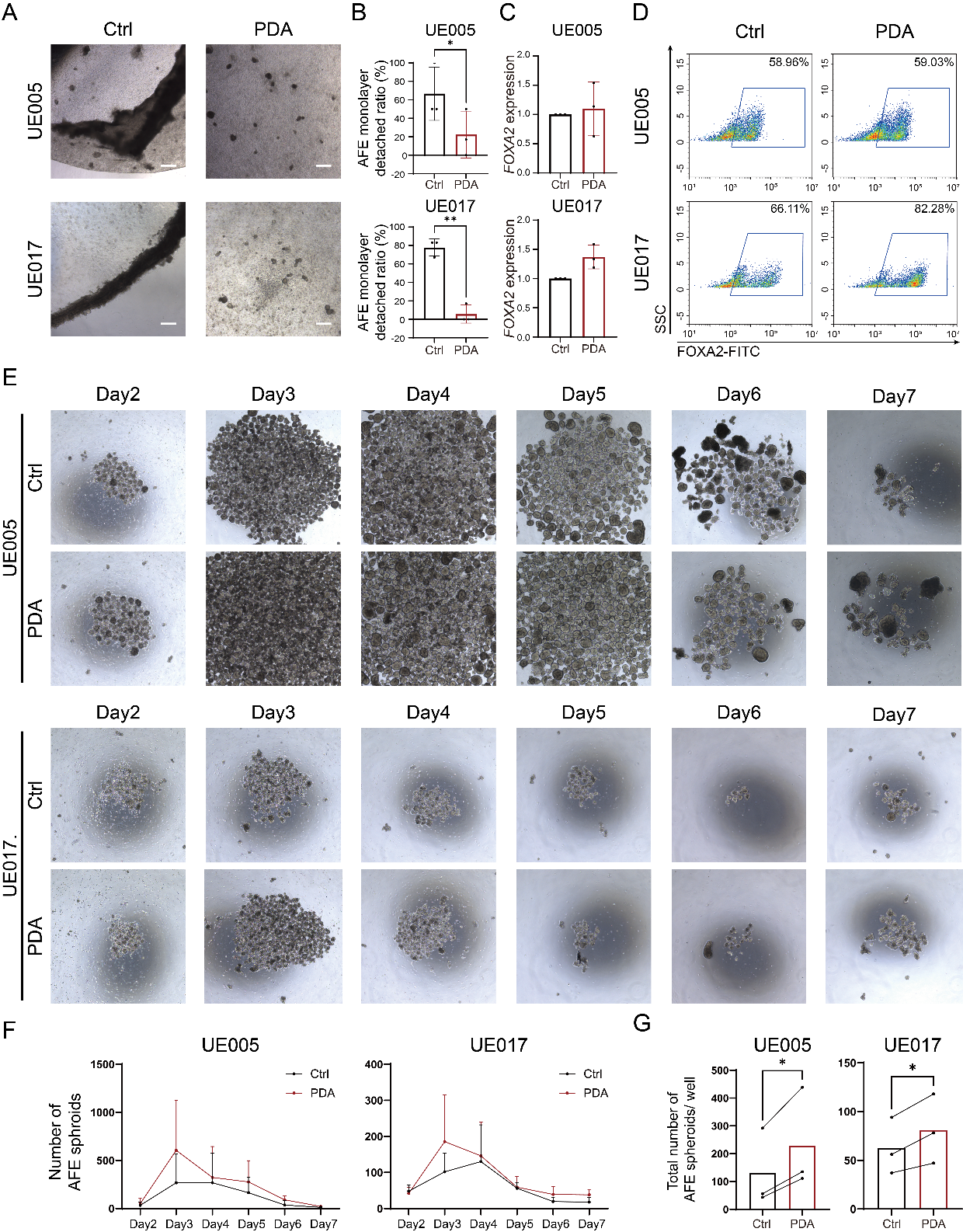


Fig.S7 PDA coating maintains hiPSC (UE005, UE017) -AFE differentiation, reduces AFE monolayer detachment and boosts AFE spheroids yield and duration. A) Representative bright-field images of AFE adherent cells on the control and PDA coating plates, scale bar: 100 µm; B) Quantification of AFE monolayer detachment ratio in different coating conditions; C) qPCR analysis of *FOXA2* expression of AFE adherent cells; D) Representative flow cytometry profiles of FOXA2 positive cells in AFE; E) Representative bright-field images of AFE spheroids collected from day 2 to day 7 of AFE stage; F) Quantitative analysis of the number of AFE spheroids from day 2 to day 7; G) Quantitative analysis of the total number of AFE spheroids. Statistical significance is indicated as follows: **P* < 0.05, ***P* < 0.01.


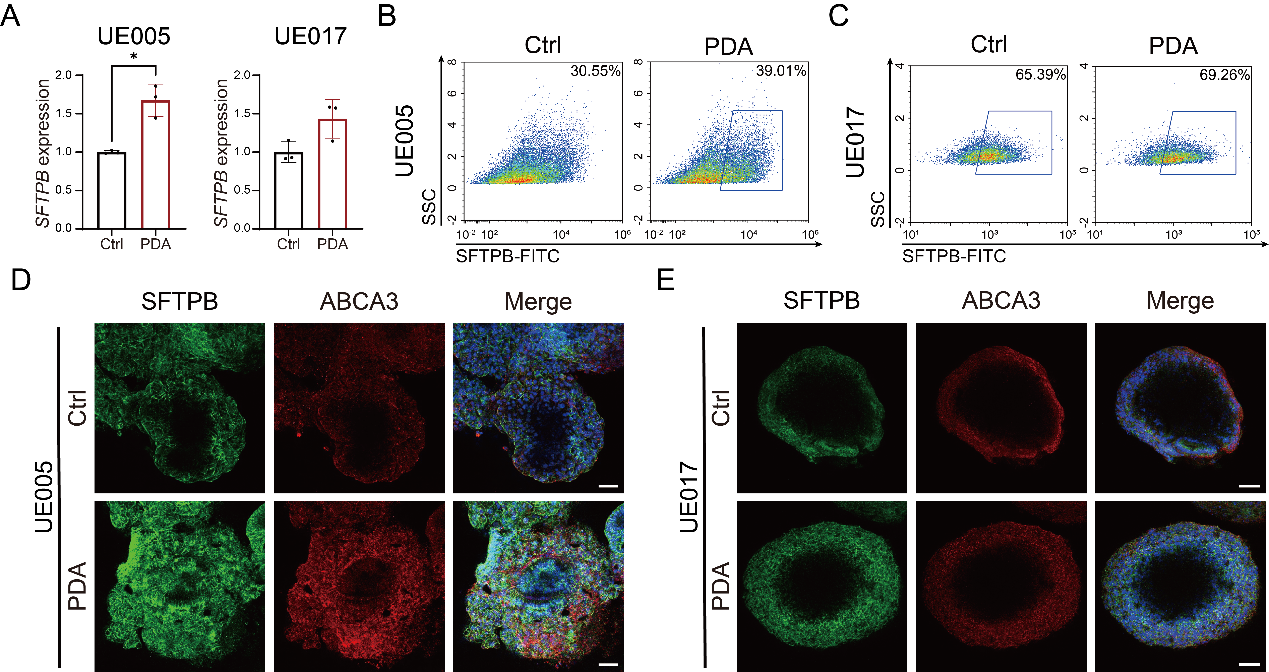


Fig.S8 PDA NPs enhances hiPSC (UE005, UE017) -AT2 maturation. A) qPCR analysis of *SFTPB* of ALOs derived from UE005 and UE017 hiPSC lines; B, C) Representative flow cytometry profiles of SFTPB positive cells in UE005 and UE017 derived ALOs; D, E) IF staining of SFTPB and ABCA3 of UE005 and UE017 derived ALOs, scale bar: 50 µm. Statistical significance is indicated as follows: **P* < 0.05.

**Supplementary Table 1. Reagents used in culture media**

| **Reagent name** | **Vandor (Cat#)** |
| --- | --- |
| MCDB131 | Thermo Fisher (Cat#10372019) |
| Advanced DMEM/F12 | Thermo Fisher (Cat#12634010) |
| DMEM/F12 medium | Thermo Fisher (Cat#10565018) |
|  |  |
| Glucose | Sigma (Cat#G7528-1kg) |
| Sodium bicarbonate (NaHCO_3_) | Sigma (Cat#6014-500g) |
| Bovine serum albumin (BSA) | Proliant (Cat#69700) |
| Penicillin/Streptomycin (P/S) | Thermo Fisher (Cat#15140122) |
| GlutaMAX | Thermo Fisher (Cat#35050061) |
| B27 | Thermo Fisher (Cat#17504044) |
| N-2 | Thermo Fisher (Cat#17502048) |
| ITS-X | Thermo Fisher (Cat#51500056) |
| HEPES | Thermo Fisher (Cat#15630080) |
|  |  |
| Activin A | StemCell (Cat#78001) |
| CHIR-99021 (CHIR) | MCE (Cat#HY-10182) |
| SAG | MCE (Cat#HY-12848) |
| SB-431542 (SB) | MCE (Cat#HY-10431) |
| Noggin | MCE (Cat#HY-P7051A) |
| FGF4 | MCE (Cat#HY-P7014) |
| DAPT | MCE (Cat#HY-13027) |
| BMP4 | MCE (Cat#HY-P7007) |
| FGF7 | MCE (Cat#HY-P70597) |
| FGF10 | MCE (Cat#HY-P70695) |
| Retinoic acid (RA) | Sigma (Cat#R2625) |
| Dexamethasone (DXMS) | MCE (Cat#HY-14648) |
| 8-Br-cAMP (8-Br) | MCE (Cat#HY-12318) |
| 3-isobutyl-1-methylxanthine (IBMX) | MCE (Cat#HY-12318) |

**Supplementary Table 2. Detailed medium formulation of hPSC-derived ALOs**

| Media | DE  Day 1 | DE  Day 2 | DE  Day 3 | AFE  Day 4-6 | LPC  Day 7-14 | ALO  Day 15-28 |
| --- | --- | --- | --- | --- | --- | --- |
| Basel media | MCDB131  Glucose ( 1.8 mg/mL)  NaHCO_3_ (1.5 mg/mL)  BSA (5 mg/mL)  GlutaMAX (1%)  P/S (1%) | | | Advanced DMEM/F12  B27 (2%)  N-2 (1%)  HEPES (10 mM)  GlutaMAX (1%)  P/S (1%) | | DMEM/F12  B27 (1%)  BSA (0.25%)  P/S (1%)  ITS-X (50 nM) |
| Add on day of use | 100 ng/mL Activin A | | | 10 μM SB  200 ng/mL Noggin  1 μM SAG  500 ng/mL FGF4  2 μM CHIR | 3 μM CHIR  10 ng/mL FGF7  10 ng/mL FGF10  20 μM DAPT  20 ng/mL BMP4  0.5 μM RA | 50nM DXMS  100nM 8-Br  100nM IBMX  3μM CHIR  10μM SB  10 ng/mL FGF7 |
|  | 3μM Chir | 0.1μM Chir |  |  |  |  |

**Supplementary Table 3. Primers used in the present study**

| **Gene** | **Forward (5’-3’)** | **Reverse (5’-3’)** |
| --- | --- | --- |
| *OCT4* | CCGAAAGAGAAAGCGAACCAG | ATGTGGCTGATCTGCTGCAGT |
|  |  |  |
| *SOX17* | GCATGACTCCGGTGTGAATCT | TCACACGTCAGGATAGTTGCAGT |
| *FOXA2* | GGGAGCGGTGAAGATGGA | TCATGTTGCTCACGGAGGAGTA |
|  |  |  |
| *NKX2-1* | CGGCATGAACATGAGCGGCAT | GCCGACAGGTACTTCTGTTGCTTG |
| *SOX2* | GCTTAGCCTCGTCGATGAAC | AACCCCAAGATGCACAACTC |
| *SOX9* | GACTACACCGACCACCAGAACTCC | CTGAGCTCGGCGTTGTG |
| *CDX2* | GGGCTCTCTGAGAGGCAGGT | GGTGACGGTGGGGTTTAGCA |
| *PAX6* | CGAATTCTGCAGGTGTCCAA | ACAGACCCCCTCGGACAGTAAT |
| *PAX8* | TGCCTCACAACTCCATCAGA | CAGGTCTACGATGCGCTG |
|  |  |  |
| *SFTPB* | TGCCTGGACCACCTCATCCTTG | GTCCTCACACTCTTGGCATAGG |
| *SFTPC* | AGCAAAGAGGTCCTGATGGA | CGATAAGAAGGCGTTTCAGG |
| *HOPX* | GCCTTTCCGAGGAGGAGAC | TCTGTGACGGATCTGCACTC |
| *AGER* | GCCACTGGTGCTGAAGTGTA | TGGTCTCCTTTCCATTCCTG |
|  |  |  |
| *GAPDH* | TGCACCACCAACTGCTTAGC | GGCATGGACTGTGGTCATGAG |

**Supplementary Table 4. Primary and secondary antibodies used in the present study**

| **Protein Name** | **Antibody** | **Vendor(cat#)** | **Application** |
| --- | --- | --- | --- |
| SOX17 | Anti-SOX17 antibody [OTI3B10] | Abcam (Ab84990) | FCM, IF (1:200) |
| FOXA2 | Anti-FOXA2 antibody [EPR4466] | Abcam (Ab108422) | FCM, IF (1:400) |
|  |  |  |  |
| NKX2-1 | Anti-TTF1/Nkx2-1 antibody [EP1584Y] | Abcam (Ab76013) | IF (1:250),  WB (1:2000) |
| SOX9 | Anti-SOX9 antibody [3C10] - BSA and Azide free | Abcam (Ab76997) | IF (1:500),  WB (1:2000) |
| SOX2 | Anti-SOX2 antibody | Abcam (Ab239218) | IF (1:50),  WB (1:2000) |
|  |  |  |  |
| AGER | Human/Mouse/Rat RAGE/AGER Antibody | R&D Systems (AF1145) | FCM, IF (1:250) |
| SFTPB | SP-B Antibody (F-2) | Santa Cruz Biotechnology (Sc-133143) | FCM, IF (1:200),  WB (1:1000) |
| ABCA3 | Anti-ATP-binding cassette sub-family A member 3 antibody | Abcam(ab99856) | IF (1:200) |
|  |  |  |  |
| β-tubulin | β-Tubulin Mouse mAb (HRP Conjugated) | Beyotime (AF2839) | WB (1:5000) |
| β-actin | Beta Actin Monoclonal antibody | proteintech(66009-1-Ig) | WB (1:5000) |
|  |  |  |  |
| Secondary antibodies | Donkey anti-Rabbit IgG (H+L) Highly Cross-Adsorbed Secondary Antibody, Alexa Fluor 488 | Invitrogen (A-21206) | IF (1:500) |
|  | Donkey anti-Mouse IgG (H+L) Highly Cross-Adsorbed Secondary Antibody, Alexa Fluor 488 | Invitrogen (A-21202) | IF (1:500) |
|  | Donkey anti-Rabbit IgG (H+L) Highly Cross-Adsorbed Secondary Antibody, Alexa Fluor546 | Invitrogen (A-10040) | IF (1:500) |
|  | Donkey anti-Goat IgG (H+L) Cross-Adsorbed Secondary Antibody, Alexa Fluor 546 | Invitrogen (A-11056) | IF (1:500) |
|  | Donkey anti-Mouse IgG (H+L) Highly Cross-Adsorbed Secondary Antibody, Alexa Fluor 647 | Invitrogen (A-31571) | IF (1:500) |
|  | Donkey anti-Goat IgG (H+L) Cross-Adsorbed Secondary Antibody, Alexa Fluor™ 647 | Invitrogen(A-21447) | IF (1:500) |
|  | HRP-Donkey Anti-Goat IgG (H+L) | proteintech (SA00001-3) | WB (1:5000) |
|  | HRP-conjugated Affinipure Goat Anti-Mouse IgG (H+L) | proteintech (SA00001-1) | WB (1:5000) |
|  | HRP-conjugated Affinipure Goat Anti-Rabbit IgG (H+L) | proteintech (SA00001-2) | WB (1:5000) |
